# Relevance of plant growth-promoting bacteria in reducing the severity of tomato wilt caused by *Fusarium oxysporum* f. sp. *lycopersici* by altering metabolites and related genes

**DOI:** 10.3389/fmicb.2024.1534761

**Published:** 2025-01-20

**Authors:** Waquar Akhter Ansari, Ram Krishna, Sarvesh Pratap Kashyap, Khalid Mashay Al-Anazi, Mohammad Abul Farah, Durgesh Kumar Jaiswal, Akhilesh Yadav, Mohammad Tarique Zeyad, Jay Prakash Verma

**Affiliations:** ^1^Department of Agriculture, Faculty of Science, Marwadi University Research Center, Marwadi University, Rajkot, India; ^2^Institute of Environment and Sustainable Development, Banaras Hindu University, Varanasi, India; ^3^Bio Zenith Scientific Research Pvt. Ltd., Mirzapur, India; ^4^Department of Zoology, College of Science, King Saud University, Riyadh, Saudi Arabia; ^5^Department of Biotechnology, Graphic Era (Deemed to be University), Dehradun, India; ^6^Department of Plant Science, University of California, Davis, Davis, CA, United States; ^7^Department of Agricultural Microbiology, Faculty of Agriculture Sciences, Aligarh Muslim University, Aligarh, India

**Keywords:** Fusarium wilt, tomato, PGPB, bio-priming, antioxidative enzyme, gene expression analysis

## Abstract

Among the biotic stresses, wilt disease severely affects tomato quality and productivity globally. The causal organism of this disease is *Fusarium oxysporum* f. sp. *lycopersici* (*Fol*), which is very well known and has a significant impact on the productivity of other crops as well. Efforts have been made to investigate the effect of plant growth-promoting bacteria (PGPB) on alleviating tomato wilt disease. Four PGPB strains, such as *Pseudomonas aeruginosa* BHUPSB01 (T1), *Pseudomonas putida* BHUPSB04 (T2), *Paenibacillus polymyxa* BHUPSB16 (T3), and *Bacillus cereus* IESDJP-V4 (T4), were used as inocula to treat *Fol-*challenged plants. The results revealed that PGPB treatments T1, T2, T3, and T4 were able to decrease the severity of Fusarium wilt in the tomato plants at different levels. Among the treatments, T3 displayed the strongest protective effect, with the lowest disease frequency, which was 15.25%. There were no significant differences observed in parameters such as fruit yield and relative water content in the PGPB-inoculated plants, although T3 and T4 showed minimal electrolyte leakage. Significant changes in chlorophyll fluorescence were also recorded. A lower level of H_2_O_2_ and malondialdehyde (MDA) was observed in the T3 and T4 treatments. In addition, proline accumulation was highest in the T3-treated plants. Antioxidative enzyme activities, such as catalase (CAT), peroxidase (POD), and superoxide dismutase (SOD), significantly increased in the PGPB-treated plants. Furthermore, the highest phenylalanine ammonia-lyase (PAL) and polyphenol oxidase (PPO) activity was reported in the T3 and T4 plants, respectively. The PGPB-treated plants showed elevated expression of the *PAL, PPO, PR3, PR2, SOD, CAT*, and *PO* genes. This study’s results reveal that PGPB strains can be utilized as biocontrol agents (BCAs) to enhance tomato resistance against Fusarium wilt.

## 1 Introduction

The tomato (*Solanum lycopersicum*) is widely cultivated worldwide due to its significant nutritional and medicinal importance, as well as its contribution to farmer’s economic prospectives (Alimohammadi et al., 2017). It provides the required nutrients for human diets, including vitamins, minerals, antioxidants, and phenolics (Zehra et al., 2023; Krishna et al., 2024). Globally, the most critical challenge that has driven researchers to develop disease-resistant strategies is the widespread impact of devastating pests and pathogens on tomato production and productivity (de Almeida et al., 2020; Pandey et al., 2021). Among the several pathogenic organisms, fungi with phytopathogenic properties play a key role in the onset, progression, and deleterious consequences of disease (Akbar et al., 2023; Singh et al., 2023). Fungal diseases have evolved over the time in several ways, seriously harms crops, and significantly affecting food security (He et al., 2020). Research aimed at deciphering the molecular mechanisms underlying the genesis of these diseases and comprehending their infection processes is of great significance.

Fusarium wilt caused by *Fusarium oxysporum* f. sp. *lycopersici* (*Fol*) stands out as a significant threat to tomatoes, whether cultivated in fields or greenhouse conditions (Ma et al., 2023; Pandey et al., 2024). During the development of vascular wilt disease, the pathogen initially penetrates through the root epidermis and connects to the root cortex by growing intracellularly and intercellularly. Finally, it establishes its growth in the root xylem vessels and then disseminates its hyphal extensions to the main root and stem of wilt-susceptible plants (Yadeta and Thomma, 2013). However, the growth of the pathogen in resistant plant species is mainly restricted to the cortex and epidermal cells. During its growth, the accumulation of fungal mycelia in the xylem vessels blocks water transport through the xylem (vessels plugging), leading to necrosis, chlorosis of aerial parts, and finally resulting in massive foliage wilting and plant death (Jiménez-Hernández et al., 2024). Economically, Fusarium wilt poses a threat to tomato crops, causing damage typically ranging from 30 to 40%, with potential losses soaring up to 80% under ideal conditions (Ghazalibiglar et al., 2016). According to a report, the economic losses due to Fusarium wilt disease were in the range of 25–55% across various regions in India (Ghazalibiglar et al., 2016).

Although the use of beneficial microbes as biocontrol agents (BCAs) is not a new approach and has been applied continuously in perennial crops for several decades (Bubici et al., 2019), this strategy has been found to have beneficial effects and positive impacts on short-term annual plants (Díaz-Gutiérrez et al., 2021). The principle objective of using beneficial microbes for plant disease management is to enhance the growth and nutrient uptake of plants, either directly or indirectly, and to alleviate both biotic and abiotic stresses. Beneficial microorganisms, commonly known as biocontrol agents (BCAs), actively inhibit the growth of phytopathogens and other antagonistic microbes, and microbial priming has the potential to stimulate the immune response ahead of encountering challenging pathogens. In addition, biocontrol microorganisms commonly utilize mechanisms such as mycoparasitism, the production of toxic and antifungal compounds (antibiosis), competition for space and essential nutrients required by pathogens, and the induction of systemic resistance in their host plants (Silva et al., 2019). Fusarium-tomato interaction is one of the model systems used to understand the molecular mechanism of pathogenesis and disease development in root-invading pathogens (Srinivas et al., 2019).

The use of PGPMs has emerged as an eco-friendly and sustainable approach to managing Fusarium wilt (Haruna et al., 2024). PGPMs, such as *Trichoderma* spp., *Bacillus* spp., and *Pseudomonas* spp., can enhance plant growth, induce systemic resistance, and suppress pathogen growth (Manzar et al., 2024). PGPMs promote plant growth by producing phytohormones such as indole-3-acetic acid (IAA), which enhances root development and nutrient uptake, and by producing siderophores that chelate iron, making it less available to the pathogen (Pandey et al., 2024). At the molecular level, PGPMs can induce systemic resistance in tomato plants by activating defense-related genes and pathways. This includes the production of pathogenesis-related (PR) proteins, activation of the salicylic acid (SA) and jasmonic acid (JA) signaling pathways, and enhancement of antioxidant enzyme activities (Abdelaziz et al., 2022; Kashyap et al., 2022).

Although our understanding of plant growth-promoting bacteria (PGPBs) has advanced significantly, there is still a major information gap regarding the precise mechanisms through which PGPB strains alter defense-related pathways in plants. PGPMs are known to cause both induced systemic resistance (ISR) and systemic acquired resistance (SAR); however, the exact molecular interactions and signaling pathways involved are still unclear. The precise genetic and pharmacological mechanisms behind these interactions must be better understood to employ PGPMs in sustainable agriculture (Manzar et al., 2024; Kashyap et al., 2022).

There is still a limited understanding of how signaling pathways and molecular mechanisms in plants challenged by Fol are affected by PGPM-mediated biopriming. This study was carried out to clarify defense signaling, physiological and biochemical modifications, and changes in gene expression arising from biopriming with PGPM. In this study, we evaluated the defense response of tomatoes against the *Fol* pathogen. We compared the expression profile of key defense genes, including pathogenesis-related (PR) genes, phenylalanine ammonia-lyase (PAL), and polyphenol oxidase (PPO), in two different situations, primed and unprimed by the PGPB strains, when the host was simultaneously inoculated with the *Fol* pathogen. In addition, we also measured the level of different antioxidant enzymes, such as catalase (CAT), superoxide dismutase (SOD), PAL, and PPO, and the activities of PR proteins (PR1 and PR3). Consequently, the current research was performed to assess changes in the expression pattern of *PR* genes and to quantify the activities of defense-related enzymes involved in cellular reinforcement and PR proteins in tomato leaves, preinoculated with PGPMs [*P. aeruginosa* BHUPSB01 (T1), *P. putida* BHUPSB04 (T2), *Paenibacillus polymyxa* BHUPSB16 (T3), and *Bacillus cereus* IESDJP-V4 (T4)], following infection by the *Fol* pathogen.

## 2 Materials and methods

### 2.1 PGPM strains and fungal pathogens

Four strains of PGPMs, *Pseudomonas aeruginosa* BHUPSB01 (GU124822), *P. putida* BHUPSB04 (GU124834), *Paenibacillus polymyxa* BHUPSB16 (GU124833), and *Bacillus cereus* IESDJP-V4 (MH362757), were obtained from the Institute of Environment and Sustainable Development, Banaras Hindu University, Varanasi. These four strains of PGPMs were revived on tryptone soya agar medium (HiMedia, India) by growing them for 2–3 d at 28 ± 2°C. These PGPM strains were selected based on their antagonistic performance against *Fusarium oxysporum* species in our earlier study (Jaiswal et al., 2022; Krishna et al., 2022; Verma et al., 2023). These strains were grown separately in tryptone soya broth (TSB) medium (HiMedia, India) at 120 rpm for 48 h at 28 ± 2°C (Krishna et al., 2022). Furthermore, the cultures were pelleted by centrifugation at 8,000 × g for 5 min, and the bacterial cells were washed once with a sterile 0.85% NaCl solution and re-suspended in it. The optical density was then adjusted to 1.0 at 600 nm (Krishna et al., 2022). The *Fusarium oxysporum* f. sp. *lycopersici* strain was cultured on potato dextrose agar (PDA) medium at 28 ± 2°C for 3–5 d. The spores of *Fusarium oxysporum* f. sp. *Lycopersici* were collected by diluting fully grown cultures up to 10^6^ spores per mL with sterile water and filtered through muslin cloth (Taylor et al., 2013).

### 2.2 Plant material

Tomato (*Lycopersicon esculentum*) seeds of the variety Kashi Adarsh were purchased from the seed sale counter of ICAR-Indian Institute of Vegetable Research, Varanasi. The seeds were initially surface sterilized in a 4% (w/v) sodium hypochlorite solution with Tween-200 (0,1%, v/v) for 10 min, then washed with sterile water, and subsequently germinated in sterile soil kept in plastic pots.

### 2.3 Experimental design and treatments

The treatments were set as follows: uninoculated control, *Fol* pathogen-challenged, T1 (*Fol-*challenged plants co-inoculated with *Pseudomonas aeruginosa* BHUPSB01), T2 (*Fol-*challenged plants co-inoculated with *P. putida* BHUPSB04), T3 (*Fol-*challenged plants co-inoculated with *Paenibacillus polymyxa* BHUPSB16), and T4 (*Fol-*challenged plants co-inoculated with *Bacillus cereus* IESDJP-V4). The *Fol* pathogen was mixed with the potting mixture at a ratio of 5 g/kg soil (Zeyad et al., 2022), which was used for transplanting 30-day-old nursery tomato plants. For biopriming with bacterial cultures, the root dipping technique was implemented, and the 30-day-old nursery tomato plants, at the time of transplanting, were dipped in 30 mL of bacterial suspension for 30 min. The control plants were soaked in a sterilized phosphate buffer for 30 min. Experiments were performed under greenhouse conditions with temperatures ranging from 20 to 25°C (day) and from 16 to 18°C (night), with a humidity of 58.4%.

Each specific pot was considered a replicate, and three biological replicates of each treatment were used, resulting in a total of nine pots. Each seedling was transplanted into a pot containing a sterile soil–sand mixture (autoclaved) in a 4:1 ratio of sand to soil. All plants under treatment were monitored carefully and watered on alternate days. The leaves from each of the three treatments were collected 30 d after nursery transplanting, washed gently with tap water, and stored at (−80°C) for further biochemical analysis.

### 2.4 Disease incidence and morpho-physiological parameters

Disease incidence (%) was assessed using the following formula:

Disease incidence (%) = (number of affected plants/total number of plants observed) × 100 (Chaterjee et al., 2021). Morphological data were recorded at the 50-day stage of the plants. For this, the root-intact plants were taken out of the potting soil, and using a measuring tape, the shoot and root lengths of the plants were recorded. Furthermore, the shoot, root, and leaf portions were separated to measure their fresh weight, while the dry mass of the same was recorded after keeping the samples at 80°C in an oven for 48 h. In addition, the number of the fruits was also recorded in triplicate by counting, and the fruit weight was measured to record the average yield per plant. We followed our previously implemented methodology for the measurements of the relative water content (RWC), electrolyte leakage (EL), and chlorophyll fluorescence (*F_v_*/*F_m_*) (Ansari et al., 2018). To measure the RWC, the fresh weight (FW) of the leaf samples was recorded, then the samples were immersed in water until full turgidity for 6 h, and the turgid weight (TW) was measured. Subsequently, the leaf samples were dried at 80°C using an oven to measure the dry weight (DW). The RWC% was calculated using the following-formula.


$$RWC\%=\left(FW-DW/TW-DW\right)\times 100$$

For the EL assessment, 1-cm diameter leaf disks were taken and dipped in 20 mL of water at room temperature for 4 h to measure the initial conductivity (EC1). The disks were then autoclaved for 30 min at 121°C to measure the final conductivity (EC2). The electrolyte leakage was measured using the following formula:


$$\mathrm{EL}\%=\left(EC1/EC2\right)\times 100$$

The *F_v_*/*F_m_* was assessed by recording the photosystem II maximum quantum efficiency using a hand-usable Plant Efficiency Analyser (Hansatech Norfolk, UK). For this measurement, 30 min of dark adaption of the leaves was carried out by employing clips on the adaxial surface. Excitation irradiance was maintained at 3,000 μmol m^−2^ s^−1^. The higher (*F_0_*) and lower (*F_m_*) fluorescence values were recorded to calculate the *F_v_*/*F_m_*:


$$F_{v}/F_{m}=F_{m}-F_{0}/F_{m}$$

### 2.5 Hydrogen peroxide, lipid peroxidation, and proline content

To measure the concentration of Hydrogen peroxide (H_2_O_2_), lipid peroxidation (LPO), and proline, we used the methodology described in previous studies (Jana and Choudhuri, 1981; Heath and Packer, 1968; Bates et al., 1973). Leaf samples weighing 0.2 gm, 0.4 gm, and 0.2 gm, respectively, were homogenized for the estimation of all these three parameters using a sodium phosphate buffer, trichloroacetic acid reagent, and sulfosalicylic acid. The absorbance of the H_2_O_2_, LPO, and proline supernatants was measured at 410 nm (UV–vis 1601 Shimadzu, Japan), 532 nm, and 520 nm.

### 2.6 Photosynthetic pigments

The chlorophyll and carotenoid content were assessed by homogenizing 0.3 g of fresh leaf samples in chilled acetone (80%). The absorbance of the supernatant at 663 nm (A_663_), 645 nm (A_645_), and 470 nm (A_470_) was measured, and the following formula was used to calculate the contents.


$$\begin{array}{l} \mathrm{Chlorophyll}\;a=\left[\left(12.7\times A_{663}\right)–\left(2.69\times A_{645}\right)\right];\\ \mathrm{chlorophyll}\;b=\left[\left(22.9\times A_{645}\right)–\left(4.68\times A_{663}\right)\right];\\ \mathrm{carotenoids}=\left[\left(1,000\times A_{470}\right)–\left(3.27\times \mathrm{chlorophyll}\;a+\mathrm{chlorophyll}\;b\right)\right]/227. \end{array}$$

### 2.7 Superoxide dismutase, catalase, peroxidase, phenylalanine ammonia-lyase, polyphenol peroxidase, and β-1,3-glucanase activity assays

The SOD activity was estimated according to the procedure by Fridovich (1974). The leaf samples (0.1 g) were homogenized in 2.0 mL of a 0.1 M phosphate extraction buffer (pH 7.8), and the absorbance of the supernatant was measured at 560 nm. Similarly, to measure the CAT activity, we followed the well-implemented methodology (McKersie et al., 1993). For the CAT estimation, 0.1 g of theleaf tissues was homogenized in a Tris HCl buffer (pH 7), and the absorbance of the supernatant was recorded. To estimate the PO activity, we used the methodology by Hammerschmidt et al. (1982). The PAL activity was determined according to the method by El-Shora (2002). For the measurements of the PAL, 0.1 g of the leaf tissues was homogenized in a Tris–HCl buffer (pH 8.8), and the absorbance of the supernatant to measure the enzyme activity was recorded at 290 nm. The polyphenol peroxidase activity was measured according to the procedure by Gauillard et al. (1993). β-1,3-glucanase assay was conducted according to the protocol by Pan et al. (1991). The leaf tissues (0.1 gm) were homogenized in a sodium acetate buffer (pH 5), and the absorbance of the supernatant was recorded at 500 nm.

### 2.8 RNA isolation, cDNA preparation, and gene expression analysis

Total RNA was extracted from the leaf tissues using a Chromous RNA isolation kit. 1 μg of the total RNA with random primers was used to synthesize the first-strand cDNA using a synthesis kit. The cDNA was further diluted and used for real-time PCR. A list of the primers used for the qRT-PCR is provided in Table 1. The first-strand cDNA (2 μL, 20 ng/μl) was used in a 20 μL reaction containing 10 μL of iQ-SYBR Green Supermix (Bio-Rad, United States), 0.8 μL of each gene-specific primer pair (100 ng/μl), and 6.4 μL of Milli-Q water. Changes in the gene expression patterns of the defense-related genes *SOD*, *CAT*, *PO*, *PAL*, *PR2,* and *PR3* were determined by qRT-PCR. The qRT-PCR was performed at 94°C (5 min) for one cycle, followed by 35 cycles at 95°C (20 s), annealing as per the Tm of each individual primer (1 min), and extension at 72°C (30 s). The output from the real-time software was analyzed and used for the cycle threshold (CT) values. Relative expression was calculated by implementing the ΔΔCT method (Livak and Schmittgen, 2001). Rubisco was used as a reference gene for normalization.

**Table 1:** List of the primers used for the qRT-PCR.

Name of gene	Primer sequence (5′-3′)
*Rubisco*	F-5′ TATCACATCGAGCCTGTTGC-3′ R-5′ AGAGCACGTAGGGCTTTGAA-3′
*SOD*	F-5′ CTGGACTTCACGGGTTTCAT-3′ R-5′ TTTGGACCGGTCAATGGTAT-3′
*CAT*	F-5′ CTCTGCCTTGACCATTGGAT-3′ R-5′ AGCATGAACAACACGCTCTG-3′
*Peroxidases*	F-5′ CCTCCAAAGAATCCGTCGTA-3′ R-5′ TTGGCTTTGAGTGCATTGAG-3′
*PAL*	F-5′ CTGGGGAAGCTTTTCAGAATC-3′ R-5′ TGCTGCAAGTTACAAATCCAGAG-3′
*PPO*	F-5′ AACCCGTTCCGTGTGAAAGTCC-3′ R-5′ –CTTCGATTACGCACCGATGCCA-3′
*PR2*	F-5′ AGACAACGTCCGAGGGTATG-3′ R-5′ TTTTTCAAGGGCCGAGTATG-3′
*PR3*	F-5′ GGTACTGCTGGTGATGATACTGCAC-3′ R-5′ GTCCTCTGCCATAGTATCTGTCTGAC-3′

### 2.9 Element quantification

The concentrations of Na, Mg, K, Ca, and Fe were measured using the 7,700 × ICP-MS system (Agilent Technologies, Santa Clara, CA, United States). The oven-dried tomato plant leaf tissues were finely powdered and subjected to microwave digestion with HNO_3_ (SupraPurTM, Merck, Kenilworth, NJ, United States). The analysis procedure followed the guidelines outlined by Bhati et al. (2014). Metal analysis was conducted on three independent replicates. Data processing was performed using Syngistix for ICP-MS (PerkinElmer, Waltham, Massachusetts, United States). Standardization of the signal responses was achieved using rhodium (Rh) as the internal standard, along with sample weights and dilution factors.

### 2.10 Statistical interpretation

The Shapiro–Wilk normality test was performed. In addition, log transformation was applied to skewed variables to enhance normality. The mean value of each replicate was used for statistical analysis, with significant differences assessed via one-way analysis of variance (ANOVA). All statistical analyzes for the present study were performed using the SPSS software (Version 16.0, SPSS Inc.). A total of three independent biological replicates were used for each experiment, with each conducted in triplicate. The mean differences were compared using Duncan’s multiple range test at a significance level of *p* ≤ 0.05.

## 3 Results

### 3.1 Disease incidence and biocontrol efficacy

To assess the induced resistance in the Fol-challenged tomato plants, the efficacy of pretreatment with PGPB strains T, T2, T3, and T4 in reducing wilt disease incidence was studied. The finding exhibited that the maximum disease incidence, reaching 87.46%, occurred in the plants subjected to the pathogen challenge. It was observed that the T3-primed tomato plants showed significantly lower disease incidence (15.25%) when compared to the Fol-challenged plants. The disease incidence was 26.2, 25.8, and 17.3%, in the T1-, T2-, and T4-treated plants, respectively.

### 3.2 Morphological parameters

The plant height of the tomato plants challenged with Fol alone and Fol + T1, Fol + T2, Fol + T3, and Fol + T4 were measured along with the control. The maximum plant height was observed in the control plants (92 cm), followed by Fol + T3 (86 cm) and Fol + T4 (82 cm), while the lowest height was observed in the case of the plants challenged with Fol alone (58 cm). The plants’ fresh and dry weight was measured, with the maximum recorded in the control plants (86 gm) and the minimum in the Fol-challenged plants (54 gm). The order of the fresh weight in the treated plants was Fol + T4 > Fol + T3 > Fol + T2 > Fol + T1. Similarly, the order of the dry weight was Fol + T2 > Fol + T3 > Fol + T4 > Fol + T1, with values ranging from 18.1 gm to 23.1 gm. The order of the root length was as follows: Fol + T3 > Fol + T4 > Fol + T2 > Fol > control> Fol + T1, with values ranging from 36 cm to 53 cm. The root fresh weight was highest in the control plants (44 gm), followed by the Fol + T3-treated plants (39.5 gm) and Fol + T4-treated plants (33 gm), while it was lowest in the plants challenged with Fol alone (21 gm). Similarly, the root dry weight was highest in the control plants (22 gm), followed by the Fol + T3-treated plants (18.9 gm) and Fol + T4-treated plants (16 gm). The leaf fresh weight and leaf dry weight were highest in the control plants (55 gm and 21.5 gm, respectively), while lowest in the case of the Fol-challenged plants (38 gm and 16 gm, respectively). Compared to the control plants, no significant difference was recorded in the leaf fresh and dry weight of the Fol + T2-, Fol + T3-, and Fol + T4-challenged plants. The total number of the fruits was highest in the control plants (18 fruits), although it was not significantly different in the Fol + T3-and Fol + T4-treated plants, both with 17 fruits. The total number of the fruits was lowest in the Fol-challenged plants (nine fruits). The total fruit weight was also highest in the control plants (730 gm), while it was not significantly different in the Fol + T3-treated plants (690 gm) and Fol + T4-treated plants (710 gm). However, the fruit weight was lowest in the Fol-challenged plants (Table 2).

**Table 2:** Effect of the PGPB inoculation on the morphological parameters of the tomato plants.

Treatment	Height (cm)	Plant fresh weight (gm)	Plant dry weight (gm)	Root length (cm)	Root fresh weight (gm)	Root dry weight (gm)	Leaf fresh weight (gm)	Leaf dry weight (gm)	Total fruit number	Fruit yield per plant (gm)
Control	92 ± 6.2^a^	86 ± 7.9^a^	23.1 ± 1.2^a^	38 ± 2.8^c^	44 ± 3.5^a^	22 ± 1.1^a^	55 ± 4.6^a^	21.5 ± 1.6^a^	18 ± 0.7^a^	730 ± 32^a^
Fol	58 ± 3.5^e^	54 ± 4.1^d^	18.1 ± 0.9^c^	39 ± 1.6^d^	21 ± 1.2^d^	13 ± 0.4^d^	38 ± 2.1^d^	16 ± 0.7^c^	12 ± 0.9^c^	380 ± 19^c^
Fol+T1	68 ± 3.1^cd^	63 ± 52^c^	19.5 ± 1.3^b^	36 ± 2.4^c^	30.6 ± 2.7^c^	14.5 ± 0.7^c^	45 ± 3.8^c^	18 ± 0.9^b^	14 ± 0.4^b^	565 ± 31^b^
Fol+T2	74 ± 4.4^c^	69 ± 3.8^b^	22 ± 1.4^a^	46 ± 2.3^b^	32.5 ± 1.7^c^	14.5 ± 0.2^c^	49 ± 2.6^b^	21 ± 1.3^a^	13 ± 1.1^b^	525 ± 31^b^
Fol+T3	86 ± 5.3^ab^	71 ± 5.7^b^	21.5 ± 0.8^a^	53 ± 1.9^a^	39.5 ± 2.4^b^	18.9 ± 1.3^b^	54 ± 3.9^a^	21 ± 1.7^a^	17 ± 0.8^a^	690 ± 34^a^
Fol+T4	82 ± 3.9^b^	73 ± 6.2^b^	20 ± 2.3^ab^	49 ± 1.7^a^	33 ± 2.9^c^	16 ± 1.2^b^	54 ± 4.9^a^	20 ± 1.2^a^	17 ± 1.3^a^	710 ± 45^a^

All values represent the means of the three replicates ± SD. ANOVA was performed, and significance was determined at the 95% confidence level. Different symbols indicate significantly different values for specific treatments (DMRT ≤ 0.05).

T1: *Pseudomonas aeruginosa* BHUPSB01, T2: *Pseudomonas putida* BHUPSB04, T3: *Paenibacillus polymyxa* BHUPSB16, and T4: *Bacillus cereus* IESDJP-V4.

### 3.3 Physiological parameters

**Figure 1:**
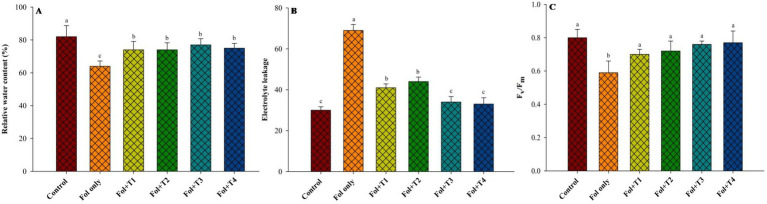
Effect of the PGPB inoculation on the **(A)** relative water content (RWC), **(B)** electrolyte leakage, and **(C)** photosynthetic efficiency (*F_v_*/*F_m_*) in the leaves of the tomato plants. All values represent the means of the three replicates ± SD. ANOVA was performed, and significance was determined at the 95% confidence level. Different symbols indicate significantly different values for specific treatments (DMRT ≤ 0.05). T1: *Pseudomonas aeruginosa* BHUPSB01, T2: *Pseudomonas putida* BHUPSB04, T3*: Paenibacillus polymyxa* BHUPSB16, and T4: *Bacillus cereus* IESDJP-V4.

The relative water content was highest in the control plants (82%), although it was not significantly different across all four treatments—Fol + T1 (74%), Fol + T2 (74%), Fol + T3 (77%), and Fol + T4 (75%). However, it was significantly lowest in the Fol-challenged plants (64%). The electrolyte leakage, an indicator of membrane damage, was highest in the Fol-challenged plants (69%), while it was lowest in the control plants (30%). The electrolyte leakage in the control and the Fol + T3-treated plants (34%) and Fol + T4-treated plants (33%) was not significantly different. Similarly, there was no significant difference in the electrolyte leakage between the Fol + T1-and Fol + T2-treated plants. The *F_v_*/*F_m_,* an indicator of the functioning of the photosynthesis system, ranged from 0.59 to 0.80, with the highest value in the control plants, followed by Fol + T4, Fol + T3, Fol + T2, and Fol + T1. It was lowest in the Fol-challenged plants (Figure 1).

### 3.4 Biochemical parameters

**Figure 2:**
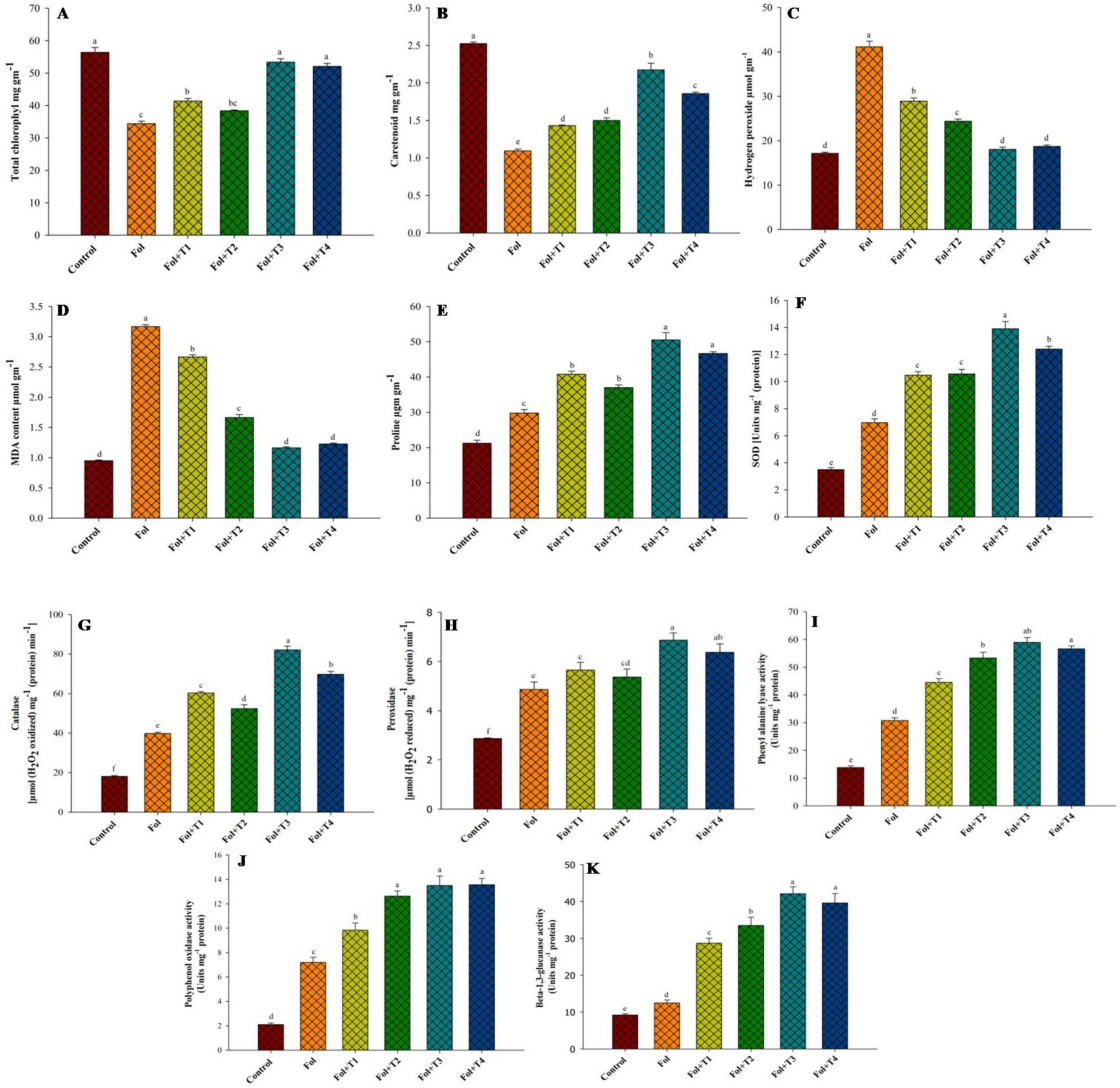
Effect of the PGPB inoculation on the **(A)** total chlorophyll, **(B)** Caretenoid, **(C)** hydrogen peroxide, **(D)** MDA, **(E)** proline, concentration, **(F)** SOD, **(G)** catalase, **(H)** peroxidase, **(I)** phenylalanine ammonia-lyase, **(J)** polyphenol oxidase and **(K)** *β*-1,3-glucanase activities in leaves of tomato plants. All the values are means of three replicates ± SD. ANOVA was found significant at the 95% significant level. Different symbols indicate significantly different values for particular treatment (DMRT ≤0.05). T1: *Pseudomonas aeruginosa* BHUPSB01, T2: *Pseudomonas putida* BHUPSB04, T3*: Paenibacillus polymyxa* BHUPSB16, and T4: *Bacillus cereus* IESDJP-V4.

The quantity of H_2_O_2_ generation was highest in the Fol-treated plants, while it was lowest in the control plants. However, it decreased in the Fol-challenged plants treated with T1, T2, T3, and T4. The Fol-challenged plants receiving T3 and T4 treatments recorded the lowest accumulation of H2O2. In comparison to the control plants, the accumulation of H2O2 in the Fol-challenged plants was 2.42 times higher. Compared to the control plants, the percent increase in the malondialdehyde (MDA) accumulation was highest (228%) in the plants challenged with Fol alone. In the Fol + T1-, Fol + T2-, Fol + T3-, and Fol + T4-treated plants, the increases were 181, 75, 22, and 29%, respectively. Similarly, the highest proline accumulation (50.5 μg^−1^ FM) was observed in the Fol + T3-treated plants, followed by the Fol + T4-treated plants (46.7 μg^−1^ FM), Fol + T1-treated plants (40.8 μg^−1^ FM), and Fol + T2 treated plants (37 μg^−1^ FM). The proline accumulation was significantly elevated in all treatments compared to the control plants. Compared to the Fol-challenged plants, the percent elevation in the proline concentration was 37, 24, 70, and 57%, respectively, in the Fol-challenged T1-, T2-, T3-, and T4-treated plants. Compared to the control plants, the total chlorophyll content was reduced in the Fol-challenged and Fol-challenged T1-, T2-, T3-, and T4-treated plants. However, the reduction in the chlorophyll content was not significant in the T3-and T4-treated plants. The lowest chlorophyll content was recorded in the Fol-challenged plants, followed by the Fol-challenged T2-and T1-treated plants. The highest carotenoid concentration was noted in the control plants (2.52 mg g^−1^), and the lowest caretenoid content was recorded in the Fol-challenged plants (1.09 mg g^−1^). Compared to the control plants, the percent reduction in the carotenoid content was 56.7, 43.3, 40.5, 13.9, and 26.4%, in the Fol-challenged, the Fol-challenged T1-, T2-, T3-, and T4-treated plants, respectively (Figure 2).

### 3.5 SOD, CAT, and POD activities

**Figure 3:**
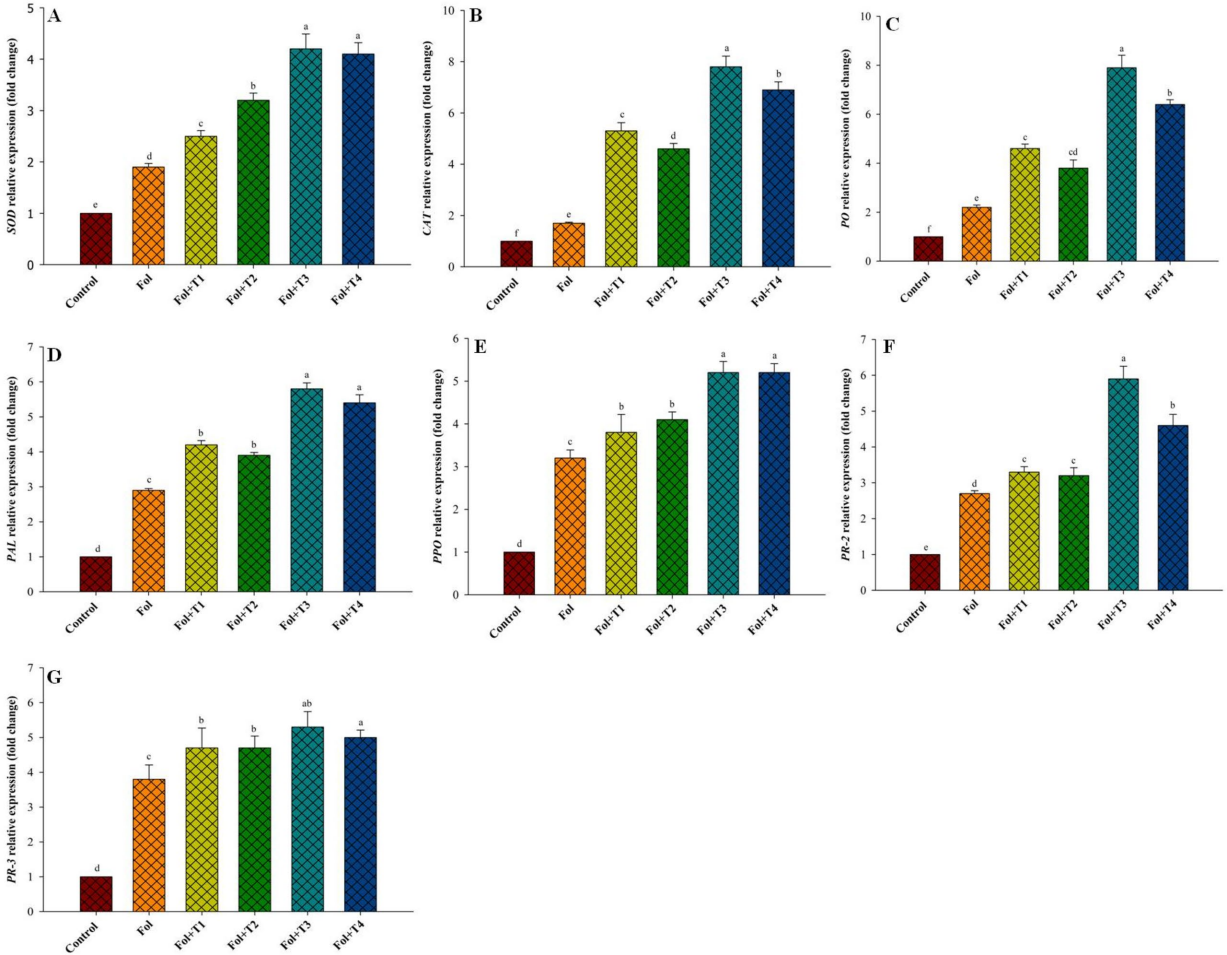
Effect of the PGPB inoculation on the **(A)** SOD, **(B)** catalase, **(C)** peroxidase, **(D)** phenylalanine ammonia-lyase, **(E)** polyphenol oxidase, **(F)** PR2 and **(G)** PR3, gene expression pattern. All the values are means of three replicates ± SD. ANOVA was found significant at the 95% significant level. Different symbols indicate significantly different values for particular treatment (DMRT ≤0.05). T1: *Pseudomonas aeruginosa* BHUPSB01, T2: *Pseudomonas putida* BHUPSB04, T3: *Paenibacillus polymyxa* BHUPSB16, and T4: *Bacillus cereus* IESDJP-V4.

The enzyme activity in the Fol-challenged and Fol-challenged T1-, T2-, T3-, and T4-treated plants increased. As shown in Figure 3, the PGPB-treated plants showed a considerable rise in the SOD levels compared to the pathogen-challenged and control plants. The SOD activity was elevated by 2.06-, 2.89-, 2.80-, 3.74-, and 3.46-fold, respectively, in the Fol-challenged, Fol-challenged T1-, T2-, T3-, and T4-treated plants. During the assays, a certain level of natural SOD activity was detected in the healthy control plants. Similarly, an increase in the catalase activity was also recorded in the Fol-challenged plants and the Fol-challenged T1-, T2-, T3-, and T4-treated plants. The levels varied, with an increase of 4.53-fold rise in the Fol-challenged T3-treated plants, followed by 3.85-, 3.33-, 2.89-, and 2.19-fold increaes in the Fol-challenged T4-, T2-, T3-treated plants, and the only Fol-challenged plants, respectively. The POD accumulation also increased in the Fol-challenged plants and the Fol-challenged plants treated with T1, T2, T3, and T4, although the rise in the accumulation was higher in the Fol-challenged plants than in the T1-, T2-, T3-, and T4-treated plants. The POD accumulation was 1.7-, 1.97-, 1.87-, 2.4-, and 2.22-fold higher in the Fol-challenged, Fol-challenged T1-, T2-, T3-, and T4-treated plants, respectively, compared to the control plants (Figure 2).

### 3.6 PAL and PPO activities

The PAL activity increased considerably in the Fol-challenged plants and Fol-challenged plants treated with T1, T2, T3, and T4 compared to the control plants. It was significantly highest in the Fol-challenged T3-treated plants, followed by the Fol-challenged T4-treated plants and Fol-challenged T2-treated plants, with respective fold increases of 4.27, 4.09, and 3.86, comparedto the control plants. However, no significant difference was observed in the PAL activity between the Fol-challenged T2-, T3-, and T4-treated plants. The lowest PAL activity was observed in the control plants. A rise in the PPO activity was noted in both the Fol-challenged plants (3.43-fold) and the Fol-challenged T1- (4.68-fold), T2- (6.02-old), T3- (6.43-fold), and T4-treated plants (6.46-fold), when compared to the control plants. However, the highest PPO activity was recorded in the Fol-challenged T4-treated plants, followed by the Fol-challenged T3-and T2-treated plants. No significant difference was observed in the PPO activity among these three treatments (Figure 2).

### 3.7 β-1,3 glucanase activity

The β-1,3-glucanase activity increased in the Fol-challenged plants and the Fol-challenged T1-, T2-, T3-, and T4-treated plants. Compared to the control plants, the fold increase in the β-1,3-glucanase activity was 1.36, 3.12, 3.64, 4.58, and 4.30, respectively, in the Fol-challenged, Fol-challenged T1-, T2-, T3-, and T4-treated plants (Figure 2).

### 3.8 Genes expression changes in the defense-related genes

The expression levels of all defense-related genes—*PAL*, *PPO*, *PR3*, *PR2*, *SOD*, *CAT,* and *PO—*increased in the Fol-challenged and Fol-challenged T1-, T2-, T3-, and T4-treated plants. The fold increase in the expression levels of these genes under the Fol-challenged condition was 2.9, 3.2, 3.8, 2.7, 1.9, 1.7, and 2.2, respectively. The highest increase in the *PAL*, *PPO*, *PR3*, *PR2*, *SOD*, *CAT,* and *PO* gene expressions was recorded in the Fol-challenged and Fol-challenged T3-treated plants, with respective fold increases of 5.8, 5.2, 5.3, 5.9, 4.2, 7.8, and 7.9, compared to the control. This was followed by the Fol-challenged T4-treated plants, which exhibited respective fold increases of 5.4, 5.2, 5, 4.6, 4.1, 6.9, and 6.4 in the gene expression compared to the control (Figure 3).

### 3.9 Ionomic responses of the Fol-challenged and Fol-challenged PGPM-treated plants

**Figure 4:**
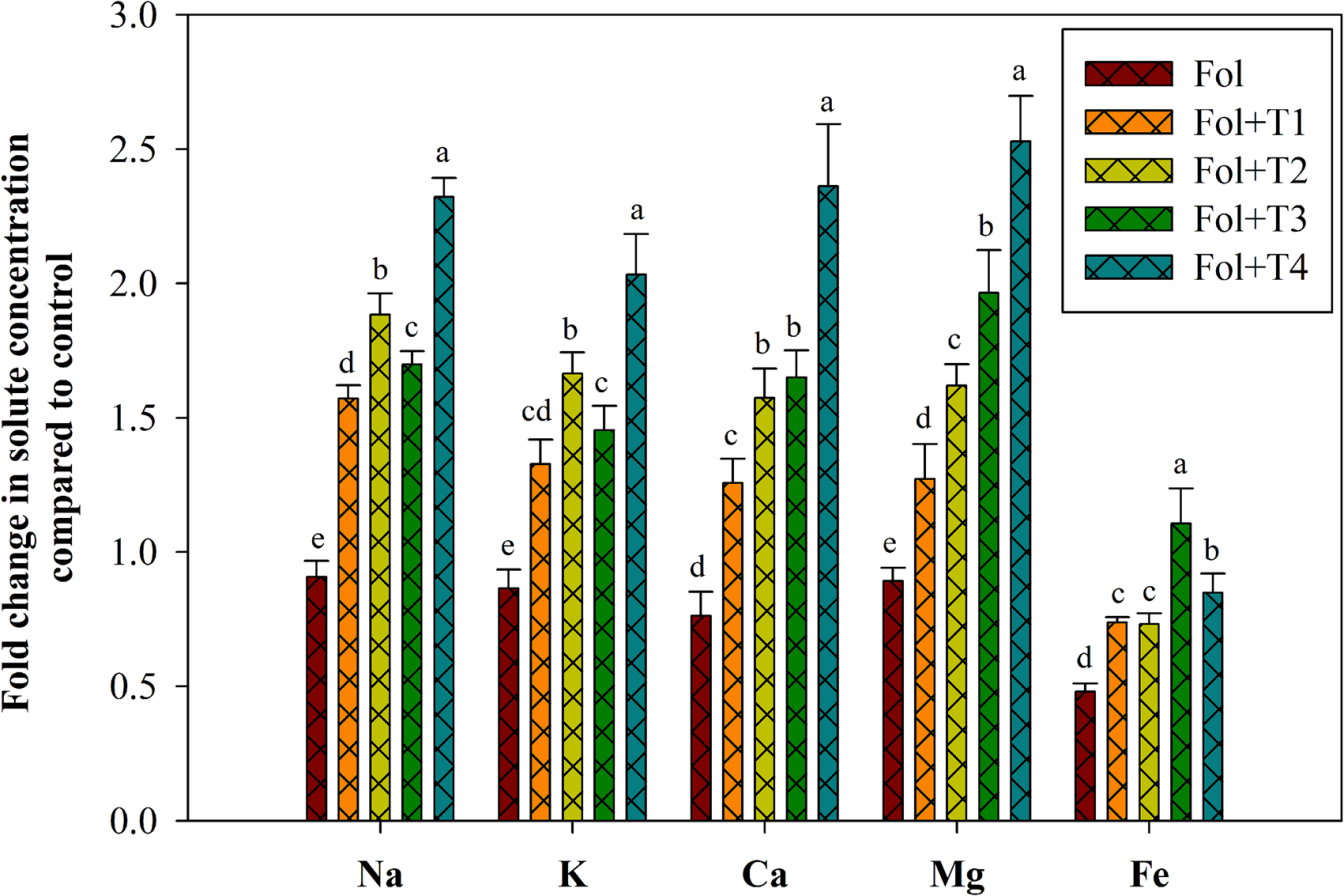
Effect of the PGPB inoculation on the concentrations of the solutes Na, K, Ca, Mg, and Fe in the leaves of the tomato plants. All values represent the means of the three replicates ± SD. ANOVA was performed, and significance was determined at the 95% confidence level. Different symbols indicate significantly different values for specific treatments (DMRT ≤ 0.05). T1: *Pseudomonas aeruginosa* BHUPSB01, T2: *Pseudomonas putida* BHUPSB04, T3*: Paenibacillus polymyxa* BHUPSB16, and T4: *Bacillus cereus* IESDJP-V4.

The concentration of the elements, such as Na, Mg, K, Ca, and Fe, in the leaves of the Fol-challenged, Fol-challenged T1-, T2-, T3-, and T4-treated tomato plants was analyzed to reveal the differential effects of the PGPB strain treatments (Figure 4). Figure 4 exhibits the alterations in the levels of the different elements in the Fol-challenged, Fol-challenged T1-, T2-, T3-, and T4-treated plants compared to the control plants. A decrease in the levels of Na, K, Ca, Mg, and Fe was recorded in the leaf tissues of the Fol-challenged plants, while a significant increase was recorded in these elements in the Fol-challenged T1-, T2-, T3-, and T4-treated plants. Maximum and significant increases in the Na, K, Ca, and Mg content were recorded in the Fol-challenged plants and the Fol-challenged T4-treated plants. However, the Fe content was significantly reduced in the Fol-challenged plants and the Fol-challenged T1-, T2-, and T4-treated plants, while a non-significant increase was recorded in the Fol-challenged T3-treated plants compared to the control plants (Figure 4).

## 4 Discussion

Initially detected in India in 1918, *Fusarium oxysporum* f. sp. *lycopersici* (*Fol*), the pathogen responsible for wilt disease, has since spread to many countries. Researchers have identified up to eight pathogenic races, highlighting the pathogen’s diverse cultural characteristics and pathogenicity (Ankati et al., 2021). Plant disease resistance can be defined based on the ability to recognize pathogenic elicitors, triggering a local immune response and initiating a rapid defense mechanism to limit the infection and prevent the spread of pathogens to nearby cells. The most common resistance responses developed by the host against pathogenic invaders include the biosynthesis and accumulation of various biochemicals (Wang et al., 2021) and the occlusion of xylem vessels through tyloses (outgrowths of xylem contact cells), which prevent the spread of pathogens (De Micco et al., 2016). The most common signaling cascades, physiological events, and biochemical changes during triggered immunity include transcriptional reprogramming of the host’s defense, activation of protein kinases, elevation of cytosolic Ca^+2^, generation of ion fluxes and reactive oxygen species (ROS) molecules, changes in gene expression profiles, and post-translational modulation of protein products, such as the phosphorylation, activation, and biosynthesis of antimicrobial compounds (El-Shora, 2002; Gauillard et al., 1993). The hypersensitive response triggered by ETI and PTI activation following the pathogen challenge leads to the increased expression of genes that encode defensive proteins, particularly pathogenesis-related proteins (PR-proteins), as well as the biosynthesis of hormones, antimicrobial peptides, and defensive enzymes and the accumulation of other defense-related secondary metabolites (Noman et al., 2019). Microbial biopriming with PGPMs results in better plant growth and development. It has been suggested that the supplementation or co-inoculation of PGPMs with plants promotes plant health through several direct mechanisms, such as plant growth promotion, increased efficiency in nutrient uptake and nitrogen assimilation, and mitigation of stress responses (Krishna et al., 2024; Krishna et al., 2022; Verma et al., 2023; Kashyap et al., 2021). However, microbial biopriming with certain PGPMs can also immune plants against successive pathogenic invaders by inducing a resistance mechanism similar to that of necrotrophs, called systemic acquired resistance (SAR).

The PGPM plays a significant role in plant growth and plant disease protection by cycling nutrients and secreting secondary metabolites and antibiotics. Odelade and Babalola (2019) and Altinok et al. (2013) reported the modulation of Fusarium wilt through biopriming with *Bacillus species* and *Pseudomonas* and *Bacillus* isolates, respectively, in tomatoes and eggplants. In a recent study, Shahzad et al. (2021) reported that the *Bacillus aryabhattai strain* SRB02 potentially reduced tomato wilt caused by *Fusarium oxysporum f.* sp. *lycopersici* (strain KACC40032). They also reported a higher accumulation of amino acids and the plant defense hormone salicylic acid (SA), along with lower levels of jasmonic acid (JA). The PGPM and its metabolites can effectively control a broad spectrum of bacterial and fungal phytopathogens (Aamir et al., 2019). Microbial biopriming is a flexible technique that can be used to strengthen a plant’s defenses against potential biotic and abiotic stressors, perhaps leading to improved tolerance or stress resistance (Zeyad et al., 2022; Aamir et al., 2019). In the present study, biopriming of the tomato plants using the PGPM strains was conducted to investigate the plant resistance mechanism in response to Fol wilt disease. Biopriming with a PGPM has been shown to have beneficial effects, including enhanced plant growth, increased nutrient uptake, improved nitrogen assimilation efficiency, and defense against various biotic stresses (Zeyad et al., 2022; Praphat et al., 2023). We found that the seedling priming of the tomato plants with the PGPM strains increased the overall growth of the tomato plants compared to the *Fol-*challenged plants. Microbial priming can also be effective in reducing disease severity and minimizing the appearance of symptoms. PGPM-induced systemic acquired resistance (SAR) and/or induced systemic resistance (ISR) play critical roles in providing resistance against various phytopathogens (Zeyad et al., 2022).

Water transport systems and membrane structures in plants are severely affected by wilt-causing plant pathogens, resulting in lower RWC (Zeyad et al., 2022; Rodrigues et al., 2018). In the current study, the implementation of T1, T2, T3, and T4 significantly enhanced the plants’ defense system, as evidenced by better RWC and lower EL. Similar findings were previously reported in maize, groundnut, and Chickpea (Zeyad et al., 2022; Ghorai et al., 2015; Fazal and Bano, 2016), using different species of *Pseudomonas* and *Bacillus*. Similarly, *F*_v_/*F*_m,_ an indicator of better photosynthetic function (Ansari et al., 2018), was higher in the T1-, T2-, T3-, and T4-treated plants, with the maximum values observed in the plants treated with T3 and T4.

In the present study, we demonstrated that biopriming with PGPB triggers complex transcriptional reprogramming, modulating the levels of defense-related transcripts. Similarly, Peng et al. (2024) reported that transcriptional reprogramming of the host’s defense genes leads to the increased expression of *PR*, *PAL,* and *PPO,* resulting in the enhanced activity of respective enzymes against the downy mildew pathogen. In our results, we investigated the enzyme activity and transcript levels of all these genes across all treatments. Biopriming with the PGPB strains led to increased enzyme accumulation and enhanced transcript levels, although these effects varied across treatments. Notably, higher enzyme activity and transcript levels were observed in the Fol + T3-treated plants. In numerous studies, it has been found that ISR induced by beneficial symbiosis is associated with the accumulation of enzymes and their respective gene expression. For example, Salas-Marina et al. (2015) demonstrated enhanced expression of *POX* and *PR* genes (glucanase) following resistance induced by *T. atroviridae* and *T. virens* against *Botrytis cinerea*, *Alternaria solani,* and *P. syringae*. Peroxidases (PO) play a crucial role in plant protection against various pathogens and also in physiological processes such as auxin catabolism, lignin biosynthesis, suberin formation, and wound healing. Sood et al. (2020) reported higher expression of *PO* in the talc-based formulation of *T. virens* (Tv1) against Fusarial wilt in tomatoes. Similarly, Singh et al. (2016) observed a 105.1% increase in PO activity after treating seeds with *T. asperellum* BHU8, compared to non-treated plants (Singh et al., 2016). Our findings align with findings and observations regarding *T. hamatum* UoM13-induced tolerance against downy mildew infection (Siddaiah et al., 2017). Furthermore, we found that the activities of the defense-related antioxidant enzymes, such as SOD, CAT, and POD, as well as their respective gene expression, were elevated in the PGPM-treated plants. Notably, the highest levels were observed in the Fol-challenged T3-treated plants compared to both the control plants and those challenged with Fol. Similarly, we also observed elevated antioxidative enzyme activity and stress-responsive gene expression in the tomato plants treated with different PGPB strains under moisture stress. This is crucial for reducing stress severity by minimizing ROS levels in the cells. The SOD activity helped counteract the harmful effects of the oxygen free radicals by transforming them into H2O2. On the other hand, under adverse conditions, the elevated CAT activity was linked to the rapid breakdown of H_2_O_2_, preventing its accumulation_._ The observation is consistent and supported by previous reports, where H_2_O_2_ was considered an indispensable factor concerned with disease tolerance against the Fol pathogen (Zehra et al., 2023). According to previous studies, POD is crucial for scavenging H_2_O_2_, and it may also act as a co-substrate in the oxidation of phenolic compounds and pathogen resistance when PAL is induced. Recent studies have highlighted the role of plant growth-promoting bacteria (PGPB) in managing oxidative stress and enhancing systemic resistance in plants. For instance, research has shown that PGPMs, such as *Bacillus* spp., *Pseudomonas* spp., and *Trichoderma* spp., can significantly reduce oxidative stress by modulating the production of reactive oxygen species (ROS) and enhancing the activity of antioxidant enzymes. This helps plants better cope with biotic and abiotic stresses (Ahmad et al., 2022).

Plants subjected to oxidative stress release hydrogen peroxide (H_2_O_2_), which serves two purposes: it can rapidly trigger antioxidant defense responses or lead to programmed cell death (Taylor et al., 2013). In the Fol + T3- and Fol + T4-treated plants, the H_2_O_2_ accumulation was lower compared to the Fol-challenged plants, suggesting a strong balance between H_2_O_2_ scavenging and H_2_O_2_ generation. The elevated MDA content in leaf tissues is considered a sign of oxidative stress that causes damage to cells (Zeyad et al., 2022; Ansari et al., 2019). The decrease in the MDA levels observed in the Fol + T1, Fol + T2, Fol + T3, and Fol + T4 treatments indicates that they may be able to reduce the peroxidation of plasma membranes during pathogen invasion, thus protecting the leaf cell membrane from damage. Similar outcomes have been reported in *Bacillus cereus* AR156-inoculated tomato plants, *Pseudomonas putida-*inoculated maize, and *Pseudomonas fluorescens-*inoculated rice (Wang et al., 2013; Nosheen and Bano, 2014; Gusain et al., 2015). In the case of plant cell protection from damage due to reactive oxygen species, proline performs a dual role—one as a scavenger and another as an osmotic balance regulator. In the current study, we found that the levels of proline increased in the Fol + T1-, Fol + T2-, Fol + T3-, and Fol + T4-treated plants. Higher proline levels in diseased plant tissues can be interpreted as a host response aimed at defending against pathogen-induced stress (Zeyad et al., 2022). Two essential leaf pigments that are used to evaluate how well the photosynthetic machinery is functioning under biotic and abiotic stress situations are chlorophyll and carotenoids (Zeyad et al., 2022; Siddaiah et al., 2017). Biotic stress often leads to chlorosis and necrosis in leaves by reducing the number of chloroplasts and breaking down chlorophyll, as noted in the present study. In the Fol-challenged plants, the levels of carotenoids and chlorophyll significantly decreased, with a less pronounced reduction in the Fol-challenged plants treated with T1, T2, T3, and T4.

When plants face biotic stresses, they often accumulate high concentrations of Na^+^ (Hasanuzzaman et al., 2020). In this context, the current study recorded the levels of Na^+^ and K^+^ to be moderately comparable in the Fol-challenged, Fol-challenged T3-and T4-treated plants, with no significant reduction observed in the shoots of the tomato plants compared to the control. The distribution of K^+^ has a significant impact on plants’ ability to withstand biotic stress, and it has been suggested that regulating K^+^ concentration is more crucial than limiting Na^+^ absorption. Biotic stress affects the uptake of several elements, including P, K, Ca, and Mg, as previously reported. In the case of the tomato plants, we observed enhanced Ca^2+^ levels in the shoots of the Fol-challenged T3-and T4-treated plants. However, the levels were reduced in the Fol-, Fol + T1-, and Fol + T2-treated plants. In plants, Ca2+ is essential for signal perception, transduction, activation of enzymes, and ionic stress mitigation. In addition to being necessary for the production of proteins and the structure of chlorophyll, magnesium (Mg^2+^) also activates many respiratory and photosynthetic enzymes. In the tomato leaves, the Mg^2+^ concentration was significantly reduced in the Fol-, Fol + T1-, and Fol + T2-treated plants, while no significant reduction was observed in the Fol + T3-and Fol + T4-treated plants. Overall, our findings show a robust correlation between the ionic condition of plants and the effects of Fol alone and Fol in combination with different strains of *Actinomycetes*.

## 5 Conclusion

This study concluded that, under the pot-based experiments, the different strains of plant growth-promoting bacteria (PGPB) primed the tomato plants and showed induced systemic resistance (ISR) against the Fusarium wilt pathogen (Fol). The elevated expression of the defense-related proteins and enzymes, such as PR proteins, PAL, PPO, and PO, confirmed effective SAR development. The enhanced *PR* gene expression and antioxidant enzyme activities corroborate previous findings, demonstrating the role of PGPB-mediated biopriming in boosting host defense. The study reported that PGPB biopriming against Fusarium wilt resulted in enhanced antioxidative enzyme levels and altered the gene expression profiles in the treated plants, highlighting the effectiveness of PGPB-based microbial biopriming against the Fol pathogen. PGPB biopriming offers an eco-friendly alternative to chemical pesticides and fertilizers, reducing environmental impact and promoting sustainable farming practices. The use of beneficial bacteria can enhance soil biodiversity and fertility, supporting long-term agricultural productivity.

## Data Availability

The original contributions presented in the study are included in the article/supplementary material, further inquiries can be directed to the corresponding authors.
